# Entomological aspects and the role of human behaviour in malaria transmission in a highland region of the Republic of Yemen

**DOI:** 10.1186/s12936-016-1179-8

**Published:** 2016-03-01

**Authors:** Samira M. A. Al-Eryani, Louise Kelly-Hope, Ralph E. Harbach, Andrew G. Briscoe, Guy Barnish, Ahmed Azazy, Philip J. McCall

**Affiliations:** Department of Vector Biology, Liverpool School of Tropical Medicine, Pembroke Place, Liverpool, UK; Department of Medical Parasitology, Faculty of Medicine and Health Sciences, University of Yemen, Sana’a, Yemen; Department of Parasitology, Liverpool School of Tropical Medicine, Pembroke Place, Liverpool, UK; Department of Life Sciences, Natural History Museum, Cromwell Road, London, UK

**Keywords:** Arabia, *Plasmodium falciparum*, *Anopheles*, *Arabiensis*, *Sergentii*, Sporozoite, EIR, Taiz, Vector, Mosquito, Surveillance

## Abstract

**Background:**

The Republic of Yemen has the highest incidence of malaria in the Arabian Peninsula, yet little is known of its vectors or transmission dynamics.

**Methods:**

A 24-month study of the vectors and related epidemiological aspects of malaria transmission was conducted in two villages in the Taiz region in 2004–2005.

**Results:**

Cross-sectional blood film surveys recorded an overall malaria infection rate of 15.3 % (250/1638), with highest rates exceeding 30 % in one village in May and December 2005. With one exception, *Plasmodium malariae*, all infections were *P.falciparum.* Seven *Anopheles* species were identified among 3407 anophelines collected indoors using light traps (LT) and pyrethrum knockdown catches (PKD): *Anopheles arabiensis* (86.9 %), *An. sergentii* (9 %), *An. azaniae*, *An. dthali*, *An. pretoriensis*, *An. coustani* and *An. algeriensis*. Sequences for the standard barcode region of the mitochondrial *COI* gene confirmed the presence of two morphological forms of *An. azaniae*, the typical form and a previously unrecognized form not immediately identifiable as *An. azaniae*. ELISA detected *Plasmodium* sporozoites in 0.9 % of 2921 *An. arabiensis* (23 *P. falciparum*, two *P. vivax*) confirming this species as the primary malaria vector in Yemen. *Plasmodium falciparum* sporozoites were detected in *An. sergentii* (2/295) and a single female of *An. algeriensis*, incriminating both species as malaria vectors for the first time in Yemen. A vector in both wet and dry seasons, *An. arabiensis* was predominantly anthropophilic (human blood index = 0.86) with an entomological inoculation rate of 1.58 infective bites/person/year. *Anopheles sergentii* fed on cattle (67.3 %) and humans (48.3; 20.7 % mixed both species), but only 14.7 % were found in PKDs, indicating predominantly exophilic behaviour. A GIS analysis of geographic and socio-economic parameters revealed that *An. arabiensis* were significantly higher (P < 0.001) in houses with televisions, most likely due to the popular evening habit of viewing television collectively in houses with open doors and windows.

**Conclusions:**

The predominantly indoor human biting vectors recorded in this study could be targeted effectively with LLINs, indoor residual spraying and/or insecticide-treated window/door curtains reinforced by education to instil a perception that effective and affordable malaria prevention is achievable.

**Electronic supplementary material:**

The online version of this article (doi:10.1186/s12936-016-1179-8) contains supplementary material, which is available to authorized users.

## Background

Malaria is the most important vector-borne disease in the Republic of Yemen, where approximately 65 % of the population are estimated to be at risk of infection and where the majority of malaria cases in the Arabian Peninsula occur [1, 2]. *Plasmodium falciparum* is widespread and responsible for more than 95 % of all cases, with the remainder caused by *P. vivax* and *P. malariae* [1, 3, 4]. The World Health Organization (WHO) reported that the number of confirmed cases fluctuates from year to year and precise estimates of incidence of malaria have been difficult to produce [1]. For example, in 2006, up to 900,000 cases and 9000 deaths were estimated to have occurred while 265,074 cases and 779 deaths were recorded in 2009 [5]. Yemen’s climate, which varies dramatically across its diverse topography, explains, at least in part, the considerable spatial and temporal variation in malaria transmission rates. At high transmission levels, malaria can be the primary cause of paediatric hospital admissions (up to 40 % in the peak malaria season), with a 0.9 % mortality rate among infected individuals [6].

In 2005, a regional strategy to eliminate malaria from the Arabian Peninsula by 2020 was launched with the support of Yemen’s National Malaria Control Programme (NMCP) [2, 5, 7]. That initiative highlighted the magnitude of malaria in the country and led to an increase in studies on malaria epidemiology [3, 4, 6, 8, 9] and a renewed focus on vector control [7]. Malaria vector control in Yemen is based on indoor residual spraying (IRS) and long-lasting insecticidal nets (LLINs) deployed alone or in combination [7]. Malaria epidemiological stratification recognizes four strata based on altitude: stratum 1 at 0–600 m; stratum 2 at 601–1000 m; stratum 3 at 1001–2000 m; stratum 4 > 2000 m, which is considered malaria free [5].

Knowledge of the natural history of the anopheline mosquitoes in Yemen and of their potential and actual roles in transmission is sparse. Fifteen *Anopheles* species have been recorded in Yemen, representing the fauna of the Afrotropical, Palaearctic and Oriental regions [10, 11]. Earlier work reported that the primary vector was the Afrotropical *Anopheles arabiensis*, which was considered to be widespread (Toffolon, 1946 unpublished, quoted in [12–14]). *Anopheles culicifacies* was recorded in the coastal plains and on the island of Socotra in the Arabian Sea, and was considered the principal vector in Al-Mahra Governorate (eastern Yemen) and on Socotra [11, 15]. *Anopheles sergentii* and the relatively uncommon *An. fluviatilis* were recorded at higher altitudes (500–1500 m) and, though unproven, both were suspected of being malaria vectors [11]. Based on these reports, the current NMCP plan [7] classifies *An. arabiensis* and *An. culicifacies* as vectors below altitudes of 600 m and *An. arabiensis* and *An. sergentii* at higher altitudes. Information on the distribution of *Anopheles* species is based on records from cross-sectional malaria surveys dating from the 1940s in published [12, 16–22] and unpublished documents [23, 24].

The first vector incrimination studies were conducted by Toffolon in 1944 and 1945 (see [12]), who reported finding sporozoites in the salivary glands of *An. gambiae* sensu lato. *Plasmodium falciparum* sporozoites were identified in *An. arabiensis* in later studies [12–14]. However, no longitudinal data exist and neither the range of anophelines present in the country nor their individual roles in malaria transmission are known. Considerable knowledge exists on the natural history, behaviour and vectorial role of the anopheline fauna in most countries that lie west of the Red Sea or east of the Persian Gulf, but extrapolation from these studies to Yemen is unlikely to be reliable, given the geographic isolation of Yemen and the unique characteristics of its arid montane habitats.

This report addresses those knowledge gaps and provides new information on the vectors of malaria, longitudinal data on entomological parameters of malaria transmission in the Taiz region and socio-cultural aspects of local communities that influence the risk of malaria. The governorate of Taiz is classed as a highly malaria-endemic area and was the location for a study on clinical and epidemiological aspects of malaria [6, 25, 26], shortly before the entomological study reported here.

## Methods

### Study sites in Taiz governorate

Located in southwestern Yemen, the territory within the governorate of Taiz extends from 200 to 2000 m above sea level (asl). The climate is sub-tropical with two wet seasons: a short season in March‒May and longer heavier rains in July‒September [11]. With few exceptions, natural watercourses are seasonal; either large streams that flow for 3‒6 months annually or smaller streams that carry water for a few weeks in the wet seasons. Natural springs, some of which are perennial, create wetlands in places. Malaria incidence increases between November and May below 1000 m and between May and September above 1000 m. Typically, transmission is unstable with outbreaks of malaria following seasonal rains [7]. To date, *P. falciparum*, *P. vivax* and *P. malariae* have been recorded [6, 11, 12, 22, 25].

**Fig. 1:**
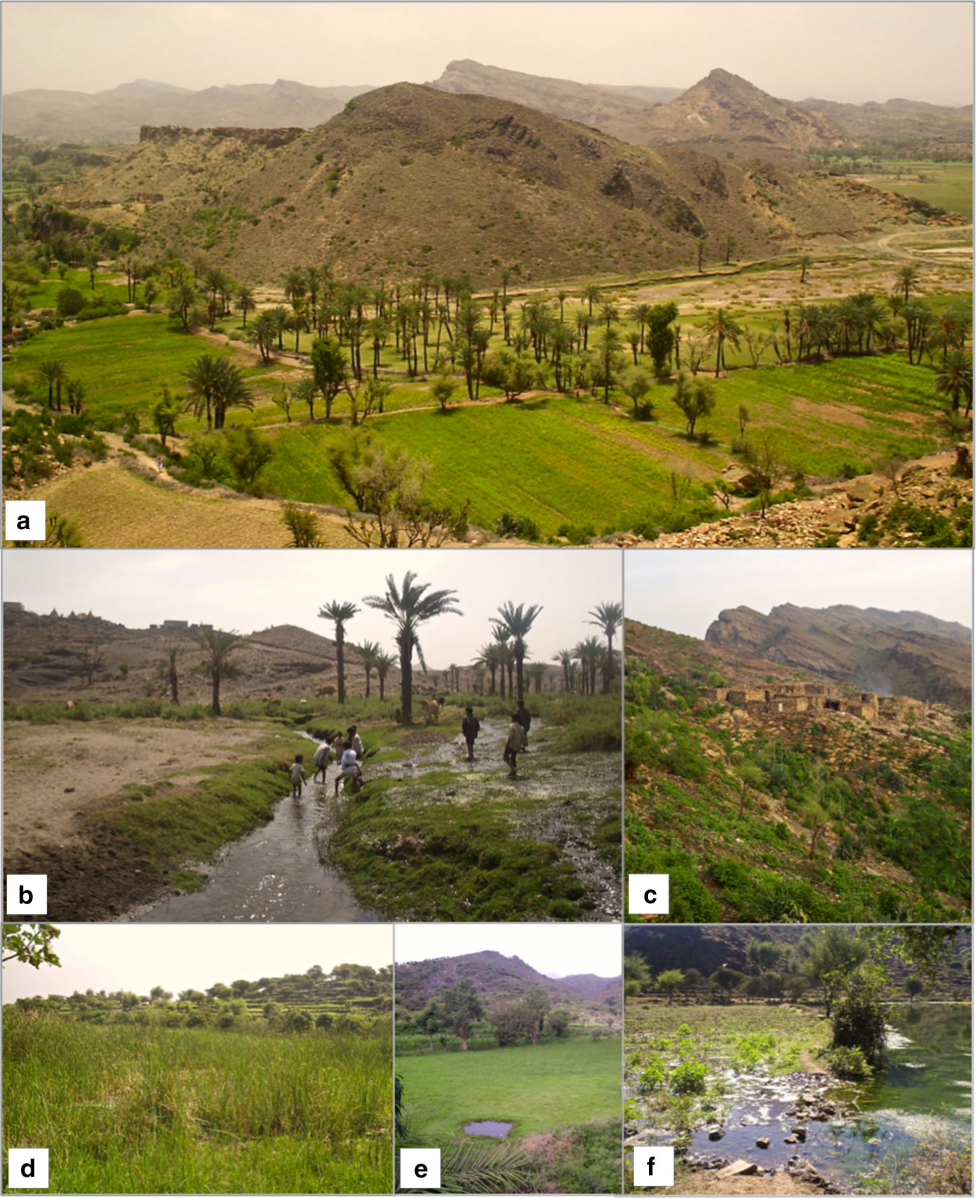
The study sites in Taiz Governorate, Yemen, during the wet season in 2004 and 2005: Ukaysh, showing the large oasis (**a**) and its main perennial stream (**b**), and a cluster of houses situated on the hill overlooking the oasis (**c**); Al-Sa’dah showing the Wadi Mae’an’ wetland (**d**, **e**) and flooded land at Wadi Al-Ahmar (**f**)

**Fig. 2:**
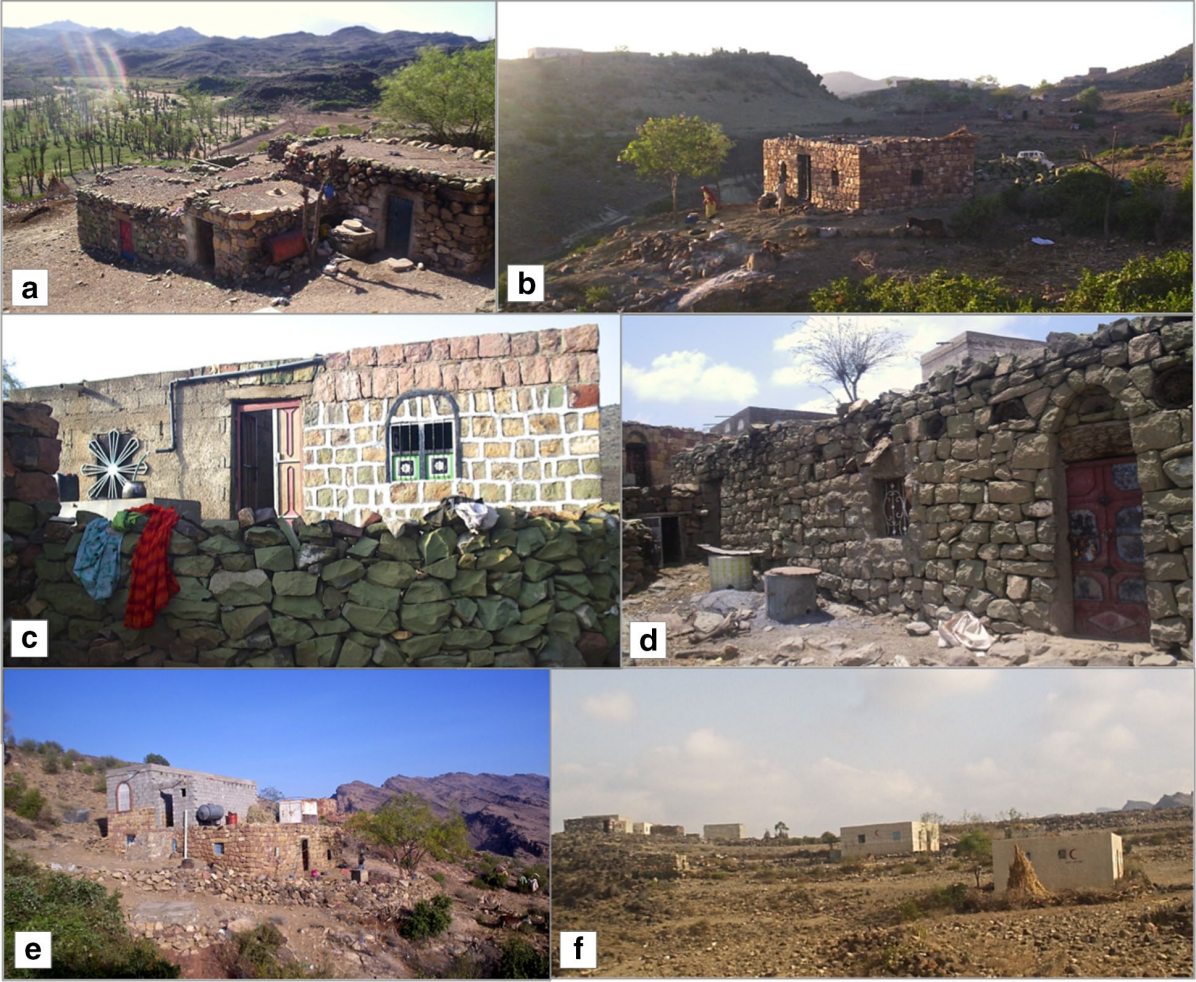
Examples of houses at the study sites: **a** Cluster of older traditional type house structures, each with a single room and no windows; **b**–**d** most common types of houses in this region, with one or two rooms and with windows; **e** two-storey house with cement block upper floor over traditional ground floor; **f** modern cement block houses with two or three rooms, known locally as ‘Helal’ houses

The geographical character of the villages in the study area is illustrated in Figs. 1, 2. The first site was at Ukaysh village (13° 39′ 12.97″ N, 43° 52′ 24.31″ E; At Taizziyah district), 910–960 m asl and 20 km northwest of Taiz city. A large (22 ha) oasis fed by a perennial stream dominates the valley (Fig. 1a–c). In the wet season, overflow creates pools even when no rain falls locally, whereas in the dry season, the villagers dig irrigation canals to divert water. The second site was Al-Sa’dah village (13°31′10.49″N 43° 56′10.79″ E; Jabal Habashy district), 1340–1400 m asl and 18 km southwest of Taiz city, with a perennial 1.8 ha wetland area (Wadi Mae’an’; Fig. 1d, e). The nearby hamlet of Wadi Al-Ahmar (13º 30′ 30.24″ N, 43º 55′ 40.68″ E; 1 km from Al-Sa’dah; see Fig. 1f) was sampled in November 2004–January 2005, following reports of a rise of malaria cases. Previously, Al Taiar et al. [6, 26] reported that malaria transmission was perennial in the Ukaysh area but limited to the wet season in Al-Sa’dah. The geographic coordinates of the aquatic habitats sampled and of all the houses at Ukaysh village where entomological, geographic and socio-economic data had been collected were recorded using a hand-held global positioning device (Magellan Meridian™ GPS, Thales Navigation).

### Human malaria infection survey

Malaria infection rates in the human populations at both Ukaysh and Al-Sa’dah were carried out by microscopic examination of Giemsa-stained thick and thin blood films. Following informed consent, finger-prick blood samples were taken from all available and consenting individuals. Infection status of all blood films was examined and confirmed by the quality assurance team at the NMCP. Anti-malarial drug treatment was provided free of charge to individuals with positive blood films, 1‒2 days after samples had been taken, following the WHO treatment guidelines provided by the NMCP.

Originally, two cross-sectional malaria surveys were scheduled for December 2004 and May 2005, coinciding with the dry and wet seasons respectively. However, 100 blood slides were stolen from the project vehicle in December 2004 and an additional survey was undertaken in December 2005 to obtain dry-season data.

### Mosquito collection

The study was undertaken in 2004 and 2005, when each site was sampled at two-week intervals over 12 months: Al-Sa’dah from January 2004‒2005, Ukaysh from March 2004‒2005. On each sampling date, adult mosquitoes were collected from nine (the maximum number possible in 1 day) randomly selected houses by pyrethrum knockdown catch (PKD) using a local insecticide aerosol (*FLEETOX*^®^; mixed pyrethroids; Arabia Felix Industries Ltd., Taiz), between 06:00 and 09:00 following WHO protocol [27, 28].

Light trap catches were performed in six different randomly selected houses using CDC miniature light traps (model no. 2836; Bioquip Products Inc., CA, USA). In these houses, all persons in sampled rooms were asked to sleep under an untreated bed net (provided by the investigator) and the trap was suspended from the ceiling with its light positioned at the level of the sleeper [29]. Householders switched the trap on before they slept, and off (closing the collecting bag) when they woke (typically at 06.00).

In June, August and December 2005 and March 2006, additional light traps were hung in animal shelters and human homes (four and two traps respectively) to investigate anopheline feeding activity on different potential hosts. Only PKD data from the longitudinal studies at both sites were used in determination of malariometric indices.

### Mosquito identification

All anophelines were identified to species using the morphological key of Glick [30]. Members of the *An. gambiae* complex were identified using a standard rDNA-PCR method [31, 32], using a protocol described previously [33]. A number of individuals could not be identified to species with certainty using morphology-based keys [30, 34, 35]. These specimens differed from females of *An. azaniae* in the absence of a presector pale spot on the wing. To examine further, DNA was extracted from the abdomens of two females confirmed as *An. azaniae* and compared with genomic DNA from one or more legs of five unidentified anopheline adult females that originated from the same sites and that were morphologically similar to the unidentified females captured during the study. Whole genomic DNA was extracted using the Qiagen DNeasy Blood and Tissue Kit (Qiagen Ltd, Sussex, England) following the manufacturers protocols. A ca. 658 bp fragment of the standard barcode region of the mitochondrial *COI* gene was amplified using the primers and thermal profiles described by Cook et al. [36]. Successfully amplified PCR products were cleaned using PCR cleanup filter plates (Merck Millipore) and subsequently sequenced bidirectionally using the same primers as for amplification. Accession numbers are available on request from the authors.

### Blood meal analysis

Individual abdomens of engorged female anophelines were crushed onto Whatman^®^ filter paper (No.3; 110 mm diameter) and stored at 4 °C in self-sealing plastic bags containing silica gel until examined by direct enzyme-linked immunosorbent assay (ELISA) [37]. Each sample was screened for human, bovine, goat and sheep blood in four separate ELISAs. Positive results were read visually 30 min after adding the substrate ABTS (2,2′ azino-bis 3-ethylbenzhiazoline-6-sulphonic acid) and hydrogen peroxide (Kirkegaard and Perry Labs, USA) in a 1:1 mixture ratio. A sub-sample of 100 positive specimens for each assay was re-run to confirm the results. All inconclusive or ambiguous results were re-tested once.

### Detection of sporozoites

Individual mosquito heads and thoraces were stored in 1.5-µl Eppendorf tubes in self-sealing plastic bags containing silica gel, for subsequent examination for *P. falciparum* and *P. vivax* circumsporozoite protein (CSP) by ELISA. A standard CDC protocol for the Antibody Sandwich ELISA method based on previously published techniques [38, 39] was used. Results were read visually 30‒60 min after adding the substrate. Positive results (visible colour in wells) were retested for confirmation.

### Climatic data

Climate data were obtained from satellite images from the IRI/LDEO Climate Data Library of the International Research Institute for Climate and Society [40]. Estimated monthly precipitation (mm), mean monthly temperature (°C) and mean monthly specific humidity (aq) were obtained from satellite data recorded by the National Oceanic and Atmospheric Administration (NOAA) and based on daily mean readings taken 2 m above the ground. This data source records specific humidity rather than relative humidity. Specific humidity measures the actual water vapour content in the air (mass of water vapour for a given mass of air expressed as grams of water vapour per kilogram of air) and is not affected by air temperature, whereas relative humidity measures the percentage of actual water vapour relative to the temperature [41].

### House survey and knowledge, attitudes and perception (KAP) survey

At the beginning of the study, data on house size and structure, numbers of persons sleeping there, presence of animals (inside/outside) the house and key possessions were recorded for all houses. Following verbal consent, information on education, mosquito prevention practices, and knowledge and perception of malaria and mosquitoes were recorded in face-to-face interviews with a senior adult member of each household, using a standard questionnaire [42] (Additional file 1).

### Data analyses

Data entered in Microsoft Excel were transferred into SPSS for Windows version 16.0. The monthly means of *An. arabiensis* were calculated by dividing the total number of PKD-collected specimens by the number of houses sampled for each month. As this was not normally distributed, values were transformed by natural log function, with a factor of one added to eliminate zero values [43]. Geometric means (GM) were calculated and 95 % confidence intervals (CI) obtained based on the assumption of normal distribution. The seasonal abundance of *An. arabiensis* for each site was plotted using the GM and the 95 % CI (upper and lower limits). Bivariate correlation was used to investigate associations between climate variables and mean monthly abundance of *An. arabiensis.* Analysis of variance (ANOVA) was used to compare more than two means.

ArcGIS software 9.1 (ArcInfo^®^ Environmental Systems Research Institute Inc., ESRI, Redlands, CA, USA) was used to produce a digital map of the study site at Ukaysh. Distances between houses, *An. arabiensis* larval habitats (distance of each house from the nearest positive site) and other key physical features within the site were measured using ArcGIS 9.1.

For spatial analyses, mean numbers of *An. arabiensis* per house were calculated by dividing the total by the number of sampling days. The Poisson skewed count data were transformed by taking the natural log of the mean number of adult female *An. arabiensis* per house (+1 to eliminate zero values). These logged mean numbers of *An. arabiensis* per house were explored for association with explanatory variables, including geographic, socio-economic and demographic factors. Distances to the nearest larval habitats of *An. arabiensis* were grouped into three categorical variables (0‒100, 101‒200 and >201 m) and the association between the variables and the abundance of *An. arabiensis* were analysed using correlation/ANOVA methods. The geometric means of *An. arabiensis* with a 95 % CI were obtained by antilog of the logged means with upper and lower limits.

### Ethical considerations

Ethical clearance for the study “Vectorial parameters of malaria transmission in Taiz, Yemen” was granted by the Faculty of Medicine and Health Sciences, University of Sana’a, and the Research Ethics Committee at the Liverpool School of Tropical Medicine (Ref 04.48).

## Results

### Human malaria infections

A total of 1724 stained blood films, were collected in December 2004, May 2005 and December 2005 at both sites, of which 285 (16.5 %) were infected. However, as 86 persons sampled (including 35 malaria positives) were not residents of either of the two study sites, they were excluded from the analysis, resulting in a malaria infection rate of 15.3 % (250/1638) at the study sites (Table 1). The highest rates of 32.4 and 31.2 % were recorded at Ukaysh in May and December 2005 respectively. However, the theft of 100 blood films at this site may account, in part at least, for the lower prevalence rate of 15.3 % recorded in December 2004. Infection rates were lower at Al-Sa’dah in all surveys, with the highest rate, 5.6 %, recorded in December 2004.

**Table 1:** Malaria infection rates during cross-sectional surveys of residents at Ukaysh and Al-Sa’dah, Taiz governorate, Yemen in 2004 and 2005

Date	No. examined	No. malaria positive	*P. falciparum*	*P. malariae*	Malaria prevalence (%)
*Ukaysh*					
2004					
December	216	33	32	1	15.3
2005					
May	306	99	99	0	32.4
December	276	86	86	0	31.2
*Al*-*Sa’dah*					
2004					
December	319	18	18	0	5.6
2005					
May	356	13	13	0	3.7
December	165	1	1	0	0.6
Total	1638	250	249	1	15.26

Infections were detected by microscope examination of Giemsa-stained blood films

*Plasmodium falciparum* comprised 99.6 % (n = 249/250) of all positive slides. *Plasmodium malariae* was detected in one blood film at Ukaysh (Table 2). *Plasmodium vivax* was not detected. During routine surveys in Ukaysh, an additional 214 blood films were examined in response to requests by villagers, of which 45.7 % (n = 16/35), 34.4 % (n = 32/93) and 43.0 % (n = 37/86) were malaria positive, in June 2005, August 2005 and March 2006 respectively.

**Table 2:** Total numbers of adult female anopheline mosquitoes collected in all sites during the study in 2004‒2005, using pyrethrum knockdown catches (PKD) (from one room in each sampled house) and light traps (in human and animal shelters)

*Anopheles* species	PKD (%)	Light traps (%)	Total (%)
*An. arabiensis*	1879 (96.4)	1082 (74.3)	2961 (86.9)
*An. sergentii*	45 (2.3)	261 (17.9)	306 (9.0)
*An. azaniae* (typical)	9 (0.5)	45 (3.1)	54 (1.6)
*An. azaniae* (atypical)	0	2 (0.1)	2 (0.1)
*An. dthali*	13 (0.7)	22 (1.5)	35 (1.0)
*An. pretoriensis*	0	30 (2.1)	30 (0.9)
*An. coustani*	2 (0.1)	14 (1.0)	16 (0.5)
*An. algeriensis* ^a^	2 (0.1)	1 (0.1)	3 (0.1)
Total	1950	1457	3407

A total of 736 PKDs in 159 houses and 330 light-trap collections in 109 households were carried out

^a^ The first record of *An. algeriensis* in Yemen

Prior to entomological sampling in Wadi Al-Ahmar of Al-Sa’dah, a malaria survey was carried out in November 2004. A total of 200 blood films were examined from this site, of which 29 were positive for *P. falciparum* (14.5 %).

### Identification and relative abundance of *Anopheles* species

A total of 3407 female anophelines were captured in light trap and pyrethrum knockdown (PKD) collections at all locations (houses and animal shelters) throughout the study (Table 2).

A sub-sample of 335 *An. gambiae s.l.* was examined using the standard intergenic rDNA-based PCR method. Of these, 317 produced a PCR band (~315 bp), and all were *An. arabiensis*.

Two female anophelines were unidentifiable morphologically. However, COI sequences obtained for five morphologically similar females collected subsequent to the surveys were subjected to an unrestricted nucleotide BLAST search for comparison with sequences available in GenBank [44]. The COI sequence obtained from these specimens shared greatest similarity with *An. funestus* isolates DQ287358, DQ287358 (93 % identity score), DQ287358, DQ287358 and DQ287358 (94 % identity score). When the sequences were compared with those obtained from two specimens of *An. azaniae*, four were found to share 98‒100 % identity and one differed by 7 %. Hence it was concluded that the two uncertain females captured during the study were atypical specimens of *An. azaniae* (Table 3).

**Table 3:** Identification of *Anopheles* adults based on comparison of their COI mtDNA sequence

Specimen	Locality	Species
♀ 40–55	Ukaysh	*An.* *azaniae* (typical)^a^
♀ 43–59	Ukaysh	*An.* *azaniae* (typical)^a^
♀ 29–52	Ukaysh	*An. azaniae* (atypical)
♂ Ukaysh 2005	Ukaysh	*An. azaniae* (atypical)
♂ 33–52	Ukaysh	*An. azaniae* (atypical)
♀ 3–59	Ukaysh	*An. azaniae* (atypical)
♀ Al Sadah tank 2004	Al Sa’dah	*An.* species?

See the “Discussion” section for morphological distinctions

^a^ Morphologically confirmed females used for comparison with the other five specimens

Seven *Anopheles* species were recorded. *Anopheles arabiensis* was the most abundant species (86.9 %) in both PKD (96.4 %; 1879/1950) and light-trap collections (74.3 %; 1082/1457). In contrast, more *An. sergentii*, the second most abundant species, were collected in light traps (85.3 %) than PKDs (14.7 %). Light trap catches also detected the presence of *An. coustani* and *An.pretoriensis* (Table 3).

*Anopheles arabiensis* and *An. sergentii* were found at all sampling sites, though numbers were higher in Ukaysh where almost 90 % (3065/3407) of the total catch was taken and all but one species, *An. coustani*, were recorded. The remaining mosquitoes were captured at Al-Sa’dah (335 individuals identified as *An. arabiensis*, *An. sergentii*, *An. pretoriensis* and *An. coustani*) and at Al-Tayyar hamlet adjacent to Ukaysh (seven mosquitoes: *An. arabiensis*, *An. sergentii*, *An. dthali* and *An.algeriensis*).

### Seasonal variation in abundance of *Anopheles* species

At Ukaysh, *An. arabiensis* was the predominant species and was recorded on all sampling dates, with sample sizes ranging from five (January 2005) to 495 (March 2005) individuals. The largest sample size for all other species was six, for *An. dthali* in December 2004.

**Fig. 3:**
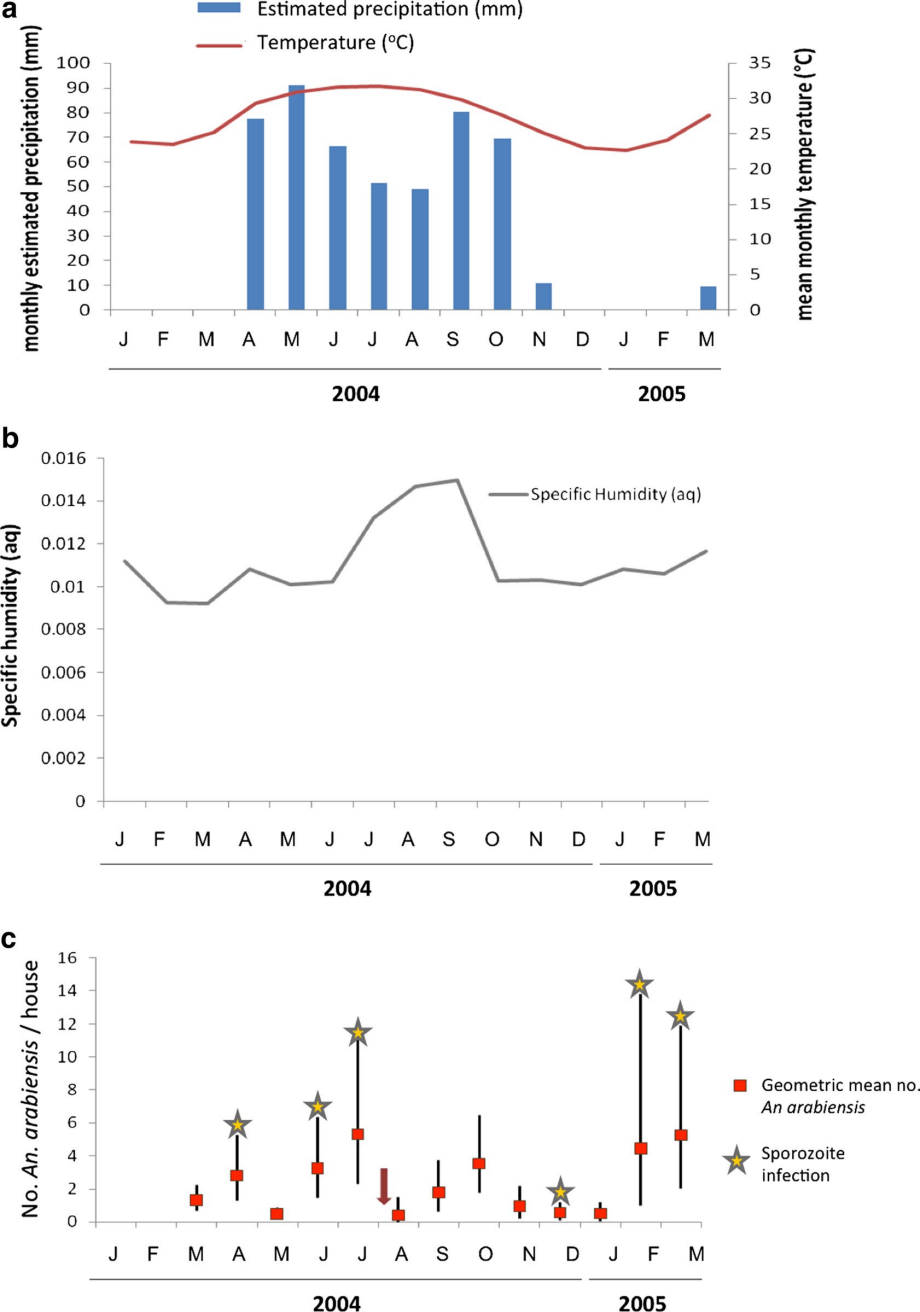
Estimated precipitation and mean temperature data (**a**) and specific humidity (**b**) at Ukaysh, Taiz governorate, Yemen from January 2004 to March 2005 (http://iridl.ldeo.columbia.edu); **c** Monthly abundance of *Anopheles arabiensis* (geometric mean number of mosquitoes per house collected by pyrethroid knockdown catch [PKD], with 95 % confidence interval) at Ukaysh (March 2004–March 2005). The *red arrow* indicates the date (1 Aug 2004) when The National Malaria Control Programme carried out larviciding at all the known larval habitats of *Anopheles* at this site

The abundance of *An. arabiensis* and associated satellite climatic variables (estimated precipitation, mean temperature and specific humidity) for January 2004‒March 2005 are shown in Fig. 3. Monthly collections were significantly different (*P* < 0.001) but there was no correlation between abundance and mean monthly temperature (*r* = 0.228; *P* = 0.453), specific humidity (*r* = 0.021; *P* = 946) or estimated precipitation (*r* = 0.085; *P* = 0.783). The National Malaria Control Programme carried out larviciding in August 2004 when all known larval habitats of *Anopheles* species at Ukaysh were treated; mosquito abundance was dramatically lower on the survey immediately after (Fig. 3).

At Al-Sa’dah, PKD samples collected between November 2004 and January 2005 comprised *An. arabiensis* (85.2 %, 127/149) and *An. sergentii* (13.4 %, 20/149). The largest PKD sample was recorded in November 2004 when 45 female *An. arabiensis* were collected in a single room.

### Identification of blood meal sources in *Anopheles* females

A total of 1233 blood-fed mosquitoes collected by all methods on all dates were screened using ELISA to identify blood meal sources (Table 4). Humans were the most common host for all mosquitoes (65.4 %), with cattle or a mixture of cattle and human blood accounting for 28 % of the blood meals. Goat blood was identified in only 2.4 % of blood meals and no mosquitoes had fed on sheep. Blood from more than one host was identified in 183 mosquitoes (14.8 %). *Anopheles arabiensis* had fed predominantly on humans (82.8 %, 946/1143), whereas 67.2 % (39/58) and 48.3 % (28/58) of *An. sergentii* had fed on cattle and humans, respectively.

**Table 4:** Identity of blood meals in adult female mosquitoes collected by pyrethrum knockdown (PKD) and light traps (LT) in houses and in animal shelters in study villages, Taiz governorate, Yemen (Jan 2004‒Mar 2005 and Mar 2006)

Mosquito species	Blood meal source										
	No. tested	Human		Cattle	Human + cattle	Goat	Goat + human	Goat + bovine	Goat + human + cattle	Sheep	Other
*An. arabiensis* (%)	1143	781 (68.3)		131 (11.5)	158 (13.8)	17 (1.5)	5 (0.4)	2 (0.2)	2 (0.2)	0	47 (4.1)
*An. sergentii* (%)	58	16 (27.6)		27 (46.6)	12 (20.7)	2 (3.4)	0	0	0	0	1 (1.7)
*An. azaniae* (typical)	13	5		5	0	1	0	0	0	0	2
*An. azaniae* (atypical)	1	0		0	1	0	0	0	0	0	0
*An. dthali*	12	2		8	2	0	0	0	0	0	0
*An. pretoriensis*	3	1		2	0	0	0	0	0	0	0
*An. coustani*	2	0		1	1	0	0	0	0	0	0
*An. algeriensis*	1	1		0	0	0	0	0	0	0	0
Total	1233	806 (65.4)		174 (14.1)	174 (14.1)	20 (1.6)	5 (0.4)	2 (0.2)	2 (0.2)	0	50 (4.1)

Of the blood meals identified from the Ukaysh longitudinal PKD data alone, 760/767 were from *An. arabiensis* and 86.0 % of those were of human origin (n = 554 from humans, 101 mixed human and bovine; 72 bovine, seven goat and 26 from unidentified hosts). Hence, the human blood index (HBI) for *An. arabiensis* was calculated as 0.862 [45]. Of 41 anopheline females analysed from Al-Sa’dah, the majority (35/41, 85.0 %) had fed on humans: *An.arabiensis* (95.0 %, 19/20), *An. sergentii* (82.4 %, 14/17), *An. coustani* (1/2), *An. pretoriensis* (1/2).

### *Plasmodium* sporozoite rates

A total of 3343 anopheline mosquitoes were screened for the presence of sporozoites, and infections were detected in 28 (0.8 %) females of three species: 25 *An. arabiensis* (*P. falciparum* and *P. vivax*), two *An. sergentii (P. falciparum)* and one *An. algeriensis* (*P. falciparum*) (Table 5a).

**Table 5:** The total numbers of *Anopheles* mosquitoes examined and sporozoite infection rates in females collected by both pyrethrum knockdown catches (PKD) or light traps (LT) between January 2004 and March 2005 at all sites, and as collected by PKD sampling only at Ukaysh and Al Sadah

*Anopheles* species	No. of females examined	*P. falciparum*	*P. vivax*	Sporozoite rate
A. All collection sites, on all dates, PKD and LT catches				
*An. arabiensis*	2921	23	2	0.9
*An. sergentii*	295	2	0	0.7
*An. azaniae*	49	0	0	0
*An. dthali*	29	0	0	0
*An. pretoriensis*	30	0	0	0
*An. coustani*	16	0	0	0
*An. algeriensis*	2	1	0	50.0
*Anopheles* sp.	1	0	0	0
Total	3343	26	2	0.8
B. Ukaysh—Longitudinal study (Mar 2004–Mar 2005) PKD data				
*An. arabiensis*	1590	18	1	1.2
*An. sergentii*	15	0	0	0
*An. azaniae*	4	0	0	0
*An. dthali*	7	0	0	0
*An. algeriensis*	1	0	0	0
Total	1617	18	1	1.2
C. Al-Sa’dah—Longitudinal study (Jan 2004–Jan 2005) PKD data				
*An. arabiensis*	125	0	0	0
*An. sergentii*	20	1	0	5
*An. coustani*	2	0	0	0
Total	147	1	0	0.7

Of the 1617 anopheline females from the 12-month longitudinal study at Ukaysh (*i.e.* PKD data only; Table 5b), 18 *An. arabiensis* were infected with *P. falciparum* and one with *P. vivax* (*Pv247*), yielding a sporozoite rate of 1.2 % (n = 19). The infected *An. arabiensis* were found in April (2/129), June (8/138) and December (1/21) of 2004, and February (2/331) and March (6/487) of 2005. The monthly sporozoite rate ranged from 0 to a high of 5.8 % in June.

At Al-Sa’dah, only *An. sergentii* was infected, with a sporozoite rate of 1.7 % (n = 2/118). The infected females were found in light trap collections in November 2004 (1/92 examined) and by PKD in January 2005 (1/20). Based on the longitudinal PKD survey, the sporozoite rate in *An. sergentii* at Al-Sa’dah was 5 % (Table 5c).

### Entomological inoculation rate (EIR)

The EIR index, a measure of the number of infective bites per person per unit time, was calculated from the human biting rate [= 0.36; the mean number of human blood-fed mosquitoes (PKD data from the longitudinal survey only = 6.7), divided by the mean number of house occupants (= 16) and multiplied by the human blood index (= 0.86)] multiplied by the sporozoite rate (= 1.2 %). The EIR for Ukaysh was calculated as 1.58 *per annum.* Thus, individuals in Ukaysh received 1.58 infective bites/person/year from *An. arabiensis* alone.

The EIR in Al-Sa’dah was lower, with transmission detected only after the rainy season, and an average of 0.003 infective bites/person/month or 0.04 infective bites/person/year.

### Demographic and socio-economic survey at Ukaysh

The total population of Ukaysh was 407 (203 males and 204 females). The median age for the female population was 13 years (range 0–80 years) and 15 for males (range 1‒80 years); 19.7 % of the total population was younger than 5 years old. Ukaysh had a total of 62 houses of four types: older traditional houses with one room and no windows, typically clustered together (Figs. 1c, 2a); the most common larger traditional houses with one or two rooms and with windows (Fig. 2b‒d) or two-storey houses with cement block upper floors over traditional ground floor (Fig. 2e); modern cement block houses with two or three rooms and windows, known locally as ‘Helal’ houses (Fig. 2f). Only nine houses (eight typical and the top rooms of one two-storey house) had window nets, most of which were damaged with holes. Typically, rooms were sealed with mud, with an earth roof and floor and a ‘lath and plaster’ ceiling; however, 23 houses (37 %) had timber ceilings.

Over 90 % (59/62) of the houses had associated animals (cattle, goats or sheep). Most houses had separate animal shelters attached to or very close to the house, but animals, especially cattle, were kept within 31.0 % (18/59) of the houses. The majority of the heads of households at Ukaysh were illiterate (86.7 %). Most of the respondents perceived malaria as a problem (96.7 %) and mosquitoes as the cause (71.7 %). Fever and vomiting were the most common (over 90 %) symptoms and signs associated with malaria, though convulsions (23.3 %) in children and coma (3.3 %) were also mentioned. The majority (96.7 %) recognized that the oasis surrounding the village was the source of mosquitoes and most (86.7 %) reported that mosquitoes bit at night all year round. The most popular local method of protection was to burn cow dung or shrubs (91.7 %), usually at the house entrance during the early evenings and prior to sleep. The second choice of protection mentioned was burning insecticide coils (30.0 %) inside the houses before sleeping. Only 18.3 % reported using insecticide aerosols.

### Risk factors for *An. arabiensis* infestation in houses at Ukaysh

Of the 62 houses in Ukaysh, 59 were sampled by PKD for adult mosquitoes during March 2004‒March 2005. *Anopheles arabiensis* were collected from 79.7 % of the houses (*n* = 1602 mosquitoes), four of which (6.8 %) yielded 56.7 % (909/1602) of the total. To investigate which parameters might have influenced mosquito entry and therefore malaria risk, associations between *An. arabiensis* infestation rates and a range of domestic, geographic and socio-economic parameters were explored.

**Fig. 4:**
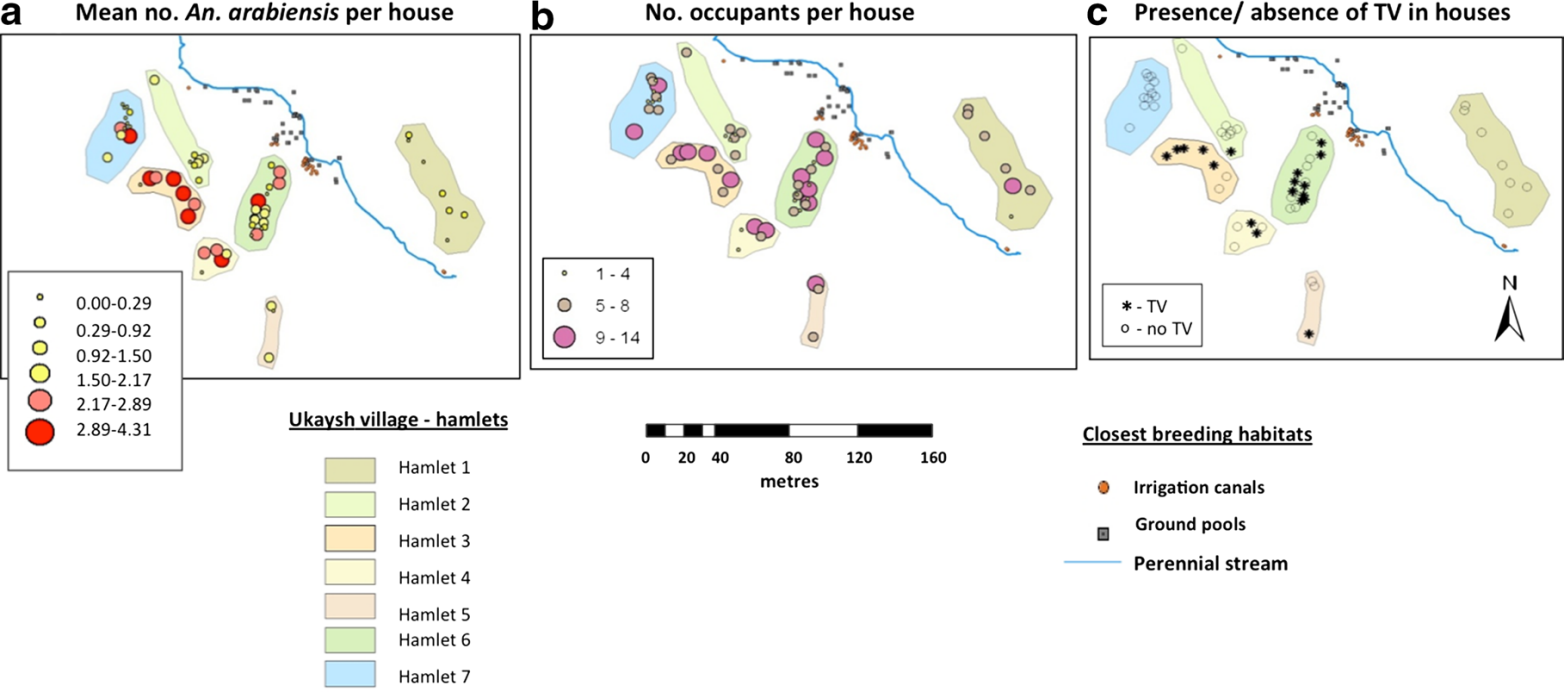
Numbers of *An. arabiensis* (log mean numbers/house) collected in houses in Ukaysh (**a**) between March 2004 and March 2005, with number of occupants (**b**) and presence or absence of televisions (**c**) in the same houses. Numbers of *An. arabiensis* were significantly higher (*P* < 0.001) in houses with televisions (5.7; 95 % CI 2.8–10.8) than those without (1.4; 95 % CI 0.8–2.1), and were positively correlated with the number of human occupants in the house (Pearson’s Bivariate correlation; *r* = 0.361; *P* = 0.005)

**Fig. 5:**
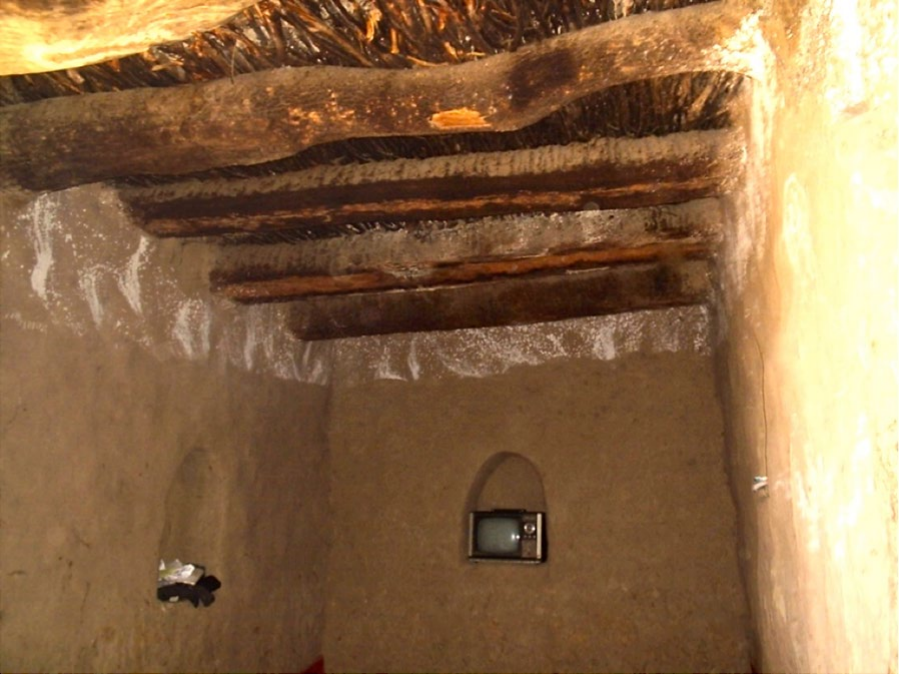
A television set within a house in Ukaysh, Taiz governorate, Yemen. Typically, up to 18 people would gather within this room every night between 19:00 and 23:00 with windows and doors left open for ventilation

The houses in Ukaysh were clustered in seven hamlets and there was a significant difference (*P* = 0.001; ANOVA) in the numbers of *An. arabiensis* caught in each hamlet, with higher numbers caught in the two ‘inner hamlets’ (Fig. 4a). The number of *An. arabiensis* was also positively correlated with the number of human occupants in the house (Pearson’s Bivariate correlation; *r* = 0.361; *P* = 0.005) (Fig. 4b). *Anopheles arabiensis* infestations differed significantly between different house types, and the number of females was significantly higher (*P* < 0.001) in houses with televisions (n = 18) (5.7; 95 % CI 2.8–10.8) than those without (n = 41) (1.4; 95 % CI 0.8–2.1) (Figs. 4c, 5).

The houses with the greatest number of *An. arabiensis*, *i.e.* in the two ‘inner hamlets’, were located approximately 200 m and more from the nearest larval habitats of *An. arabiensis* (mainly simple irrigation canals and ground pools). Houses located closer to the positive habitats (at the perennial stream), at 0‒100 m and less than 200 m, had lower numbers of *An. arabiensis*.

Infestation rates were not significantly different between homes that did or did not house animals overnight (*P* = 0.674). There were no differences in numbers of mosquitoes found in houses with different internal construction (*i.e.* houses with ‘lath and plaster’ or ‘wood/timber’ ceilings; *P* = 0.346). The numbers of *An. arabiensis* resting inside the houses was not significantly different in houses with or without window netting (*p* = 0.641), though it was noted that the window nets were usually damaged with holes and therefore would not have prevented mosquitoes from entering. Vector infestation rates were not influenced by the education level of the head of the household (*i.e.* the respondent in KAP survey; *P* = 0.881) or the socio-economic index (number of items owned) by the household (*P* = 0.117).

Finally, the use of mosquito repellents by the locals (*e.g.* burning dung or vegetation to produce a repellent smoke), whether used alone or in combination with mosquito coils or domestic insecticide aerosols, did not affect mosquito entry and there were no significant differences between these groups and those houses that did not use any form of repellent (*P* = 0.143).

## Discussion

This study is the first comprehensive longitudinal study of malaria transmission conducted in Yemen. Adult females of seven anopheline species (*An. arabiensis*, *An. sergentii*, *An. dthali*, *An. azaniae*, *An. pretoriensis*, *An. coustani* and *An. algeriensis*) were found within houses in the governorate of Taiz, one of the principal malaria-endemic areas in Yemen. *Anopheles arabiensis* and *An. sergentii* were infected with human plasmodial parasites and occurred in sufficiently high numbers to conclude that they are the main malaria vectors. Malaria sporozoites were found also in a single *An. algeriensis* female, but the scarcity of this species suggests it is unlikely to be of importance in transmission, at least in the study area.

*Anopheles arabiensis* was by far the most abundant mosquito species, comprising 86.9 % (n = 2961/3407) of the total adult female anophelines present in both PKD and light-trap collections. This was the only member of the *An. gambiae* complex found in the study area, confirming previous studies by Townson et al. [46]; from a museum specimen] and by Al-Sheikh [14]. The latter study identified mosquitoes from the Tihama area bordering Saudi Arabia, where this species was also the most prevalent anopheline.

High numbers of *An. arabiensis* dominated (96.4 %) the PKD collections, a method that selectively collects endophilic species. Clearly it is the dominant anthropophilic species in the study area. Although outdoor collections were not made, the number of anopheline females captured indoors clearly demonstrates a strong endophilic behaviour of *An. arabiensis* in this region, as in Africa [47–50] and confirms previous records from the Taiz [24] and Tihama areas of Yemen [13, 14]. However, additional exophagic or exophilic behaviour cannot be excluded since the collection methods used would not have captured such mosquitoes. Consequently, while a high degree of contact took place between *An. arabiensis* and humans indoors, it cannot be concluded that *An. arabiensis* was solely endophagic and endophilic in this region, and further studies are needed. *Anopheles arabiensis* exhibits exophilic behaviour in many regions of Africa [51–56], and both endophily and exophily may occur in the same population [50, 57–60], either as a ‘natural’ behaviour or as a selected response to indoor residual spraying [61].

The HBI of *An. arabiensis* was 86.2 % (all blood meals containing human blood; 72.9 % for human blood alone). The high HBI recorded here corresponds with those recorded in many studies in Africa. Various studies have recorded HBI levels up to 86 % (north eastern Tanzania and Malawi [49, 56]), 83.4 % (eastern Sudan [48]) and 82 % (central Sudan [62]) In Africa there can be marked variation in HBI in different areas. A very low HBI (0.23) was found in Kenya, where large numbers of cattle were present [58], whereas in another study near Kisumu in western Kenya, none of the *An. arabiensis* screened had fed on humans, although 0.6 % were *P. falciparum* sporozoite-positive [52], implying predominantly zoophagic behaviour. Conversely, in the Gambela area of Ethiopia, where there were no cattle or other livestock, *An. arabiensis* fed entirely on humans [63]. High HBI values of 0.99 were found in Mozambique [64], 0.923 in southern Zambia [65] and 0.911 on the Kenyan coast [66].

While the proportion of negatives or unidentified blood meals (4.1 %) recorded in this study might be due to feeding on non-human hosts, the likelihood that many blood meals had degraded before processing is high. The very low humidity in the study sites meant that approximately 4 % of the captured mosquitoes had dried before or during storage (or indeed, some of the blood meals may also have degraded during transfer from the field to the laboratory), a phenomenon that has been recorded in other studies [50, 67].

*Anopheles arabiensis* females were present in the study area throughout the collection period, although fluctuations in abundance occurred at each study site. At Ukaysh, where this species was most common, there was a significant difference between the monthly collections (*P* < 0.001). Two peaks were observed: one in the hot summer (June‒July) and another in the warm spring (February‒March) (Fig. 3c). There was no correlation between the monthly abundance of *An. arabiensis* and climate. Previously, Al-Maktari and Bassiouny [13] recorded peaks of *An. arabiensis* in the months of March, July and August in Zabid district, Al Hudaydah. The increase in densities of *An. arabiensis* during February and March in Ukaysh most likely resulted from a combination of warmer temperatures during these months and the seasonal construction of many irrigation canals for crops from the main stream in the village, which created more larval habitats. Such increases in numbers of *An. arabiensis* have been associated with irrigation canals in Tanzania and Kenya [52, 56, 68].

Although *An. sergentii* was the second most abundant *Anopheles* species encountered in this study, it comprised only 9.0 % (n = 306/3,407) of all anophelines collected. *Anopheles sergentii* has been found in most regions of Yemen except coastal areas [11, 13, 14, 18, 19, 23, 24]. In this study, over 85 % of *An. sergentii* were collected in light traps (n = 261/306) rather than by PKD at both study sites (39 and 6 % at Al Sa’dah and Ukaysh respectively). The presence of *An. sergentii* in light-trap collections and comparatively low numbers in PKD collections indicate the relatively uncommon combination of endophagic and exophilic behaviour. This is surprising as, given the arid nature of the region, endophily might be expected. *Anopheles sergentii* was reported to seek resting places in “caves and fissures” in hills in Jordan [69] and clearly its resting sites should be investigated further. Light traps have been effective for collecting exophilic *An. sergentii* at low densities in southern Iran [70].

The HBI of *An. sergentii* was 48.3 % (from 58 specimens collected by PKD and light traps during the entire study), with a higher proportion of bovine blood meals (46.6 %) than *An. arabiensis* (Table 4). *Anopheles sergentii* is known to be zoophilic [67, 71, 72].

*Anopheles azaniae* is of interest here as the atypical females (Table 3) were not readily identifiable using available identification keys [30, 34, 35]. A single specimen analysed had a mixed human/bovine blood meal and therefore must be considered anthropophilic and a potential malaria vector. Further investigation of *An. azaniae* is needed to determine the relative abundance of the two forms, their distribution and vectorial status, particularly since larvae of the atypical form were found in water tanks adjacent to houses.

The atypical form of *An. azaniae* differed from the typical form in lacking a presector pale spot on the wing. Other distinctions included the absence of small pale spots at the bases of veins R_4+5_, M_1+2_ and M_3+4_ (adjacent to the mediocubital crossvein, morphological terminology of Harbach and Knight [73], which are present in *An. azaniae*, and the presence of a weakly developed presector pale spot on the costa of two of four females. Unlike the atypical specimens of *An. azaniae*, which have three costal pale spots (sector, subcostal and apical) that separate four dark areas, the wings of the specimen with a 7 % difference in COI sequence had only two costal pale spots (subcostal and preapical) separating three dark areas, which led to the couplet that distinguishes between subgenera *Anopheles* and *Cellia* in the key of Glick [30]. The specimen also differed from the typical and atypical forms of *An. azaniae* in having no pale spots on the wings other than the sector pale (on the costa only) and the subcostal pale (on the costa and radius-one), and also in being a slightly larger and paler mosquito. These significant differences suggest that the specimen is likely to be a member of a species new to science.

Sporozoites of *P. falciparum* and *P. vivax* were identified in 28 mosquitoes of three anopheline species. *Plasmodium falciparum* was the predominant species (92.9 %, 26/28). *Plasmodium vivax* was only detected in two *An. arabiensis* females (Table 5a): one with *Pv210* and the other with *Pv247*. *Anopheles arabiensis* was the primary vector with an overall CS rate of 0.9 % (25/2921), of which 23 were *P. falciparum* and two were *P. vivax*. A higher CS rate of 1.2 % (19/1590), of which 18 were *P. falciparum* and one was *P. vivax*, was calculated from the PKD collections carried out at Ukaysh during March 2004‒March 2005. Previously in the Tihama area, slightly lower sporozoite rates of 0.65 % [14] by PCR and 0.7 % by salivary gland dissection [13] were recorded. This rate is lower than in Africa where sporozoite rates in *An. arabiensis* can range from 2 % [49, 74, 75] to over 7 % [76]. However, sporozoite rates similar to that detected in the present study have been recorded for this species in Eritrea (0.54–1.3 %; [77]), Ahero in western Kenya (1.1 %; [52]) and in eastern Sudan (1.4 %; [48]). Indeed, a very low sporozoite rate of 0.38 % was found in the semi-urban region of Ifakara town, Tanzania [78]. These variations may be due to different feeding behaviour of *An. arabiensis* in the different regions. Sporozoite rates are lower when the vector is more zoophilic and when other more efficient vectors are also present [79]. Moreover, ELISA and PCR have been shown to be more sensitive than salivary gland dissection [80].

*Anopheles sergentii* had a CS rate of 0.7 % (2/295). Until now, this species was only suspected to be a secondary vector in Yemen based on high densities reported during malaria outbreaks. In the Asir region of neighbouring Saudi Arabia, it is a known vector of malaria [81]. The sporozoite rate found in *An. sergentii* in this study is similar to that found in Egypt for *P. falciparum* (1.35 %; [82]) and *P. vivax* (1.2 %; [83]).

It is interesting that one of three females of *An. algeriensis* collected in the present study was infected with *P. falciparum* sporozoites. This is the first record of this species in Yemen, which is at the south-eastern edge of its known range that includes the Middle East, northern Africa and Europe from the Mediterranean to Ireland [84]. It was recorded as a potential vector during an outbreak of malaria in Libya [85]. In a recent report on vector control in the Middle Eastern Region during the 1991 war with Iraq, *An. algeriensis* was regarded as a potential vector in the Arabian Peninsula and southern Iraq [86].

The entomological inoculation rate (EIR) was calculated only from the PKD collections conducted during the longitudinal study. In Ukaysh, the villagers received 1.58 infective bites/person/year from *An. arabiensis*, the only vector at this site. Higher EIR values typically are recorded in malaria-endemic African countries where this species and others of the *An. gambiae* complex and *An. funestus* are vectors, although great variation can occur. Hay et al. [87] reviewed EIR values in the African continent where a mean annual EIR of 121 infective bites per person per year was calculated, but with a range of 0–884. Rural regions have a mean EIR of 146 (0‒884), areas with irrigated rice surroundings had a lower mean of 99 (0‒601) and those in urban areas showed the least exposure with a mean of 14 (0‒43) [87]. Lower EIRs were found in rural parts of the Gambia, ranging from 0.44‒11.15 [88].

Inoculation rates can also vary annually. In southern Zambia, following prolonged drought in 2004‒2005, Kent et al. [65] recorded zero transmission but the following year, the EIR was 1.6 and 18.3. In different ecological zones in Eritrea, EIRs for *An. arabiensis* between 0 and 70 infective bites/person/year have been recorded [89]. The method of estimating EIRs and the heterogeneity of transmission within each site have been shown to yield geographically significant differences in the intensity of malaria transmission across countries of sub-Saharan Africa [90].

With the use of GIS, the abundance of *An. arabiensis* within an endemic village in Yemen showed that the presence of televisions brings villagers together, thus rendering houses with televisions more attractive to *An. arabiensis.* Most of the houses with televisions had unprotected windows, and although windows were well fitted, nylon screens, if present, were usually badly torn, permitting mosquito entry. Such screens would achieve little anyway, since most houses leave the doors open for ventilation when rooms are crowded with people watching television.

Although nocturnal biting cycles were not investigated in this study, and further research is needed to identify the circadian biting peaks of *An. arabiensis* and *An. sergentii*, the results provide useful information for making informed decisions about vector and malaria control in the region. For example, the practice of watching television throughout the first half of the night, and the exophilic behaviour of *An. sergentii*, present a challenge. Insecticide-treated bednets (ITNs) would not be effective in households with televisions and alternatives would have to be considered. Entering mosquitoes might be reduced with insecticide-treated curtains (ITCs), which have been shown to reduce malaria transmission in Burkina Faso [91, 92]. Impregnated curtains could be fitted on windows and doors and could possibly prevent mosquitoes from entering these houses while the owners and neighbours watch television. Most of the windows and doors are small; therefore, it is feasible to fit insecticide-treated curtains. Indeed, although these social activities take place at night, they expose humans to anopheline mosquito bites much as diurnal activities expose people to the bites of *Aedes aegypti* in and around homes. Given that ITCs hung in homes have been shown to impact *A. aegypti* [93, 94], the possibility that similar interventions might be successful should be considered. However, no curtains were seen in the villages, where they would undoubtedly be considered a luxury item, unless the health benefits were to be promoted. With numerous small rooms and large families, villagers might accept these curtains more readily than ITNs, although ITNs would be suitable for houses without televisions, those with fewer family members and those where residents sleep outdoors during the hot weather. However, the introduction of ITNs and thus ITCs, as shown by the KAP survey, would probably be more acceptable in Ukaysh where the mosquito problem exists throughout the year, and perhaps less so in Al-Sa’dah where mosquitoes appear only following the rainy season. Not surprisingly, the respondents of the KAP survey conducted in Ukaysh stated they were more willing to use and pay for a bednet than those at Al-Sa’dah.

Both ITNs and ITCs would be expected to impact also on the exophilic *An. sergentii*. However, given the high proportion *An sergentii* females that had fed on cattle (67.3 %), treatment of cattle with pyrethroids, which has been shown to be effective against *An. arabiensis* [95, 96] and for reducing malaria transmission [97], might be considered.

Indoor residual spraying, while effective against endophilic mosquitoes such as *An. arabiensis*, would not impact on the exophilic *An. sergentii*. Moreover, most houses have mud inner surfaces, which may absorb insecticide and shorten the duration of its effectiveness. Indeed, some types of mud may break insecticides down chemically [98], further limiting the potential of this approach.

Most importantly, it is advisable to promote health education advice regarding malaria diagnosis, treatment, prevention and control, information on mosquito behaviour and the methods of control that will be applied eventually in Yemen, as part of the ventures jointly launched by the United Nations Environmental Programme, the Global Environment Facility and the WHO-Eastern Mediterranean Region [99], to ensure optimization of community acceptance and compliance, essential pre-requisites for successful malaria control.

## Additional file

10.1186/s12936-016-1179-8 House survey and knowledge, attitudes and perception (KAP) survey.
