# Supplementary material for: Entomological aspects and the role of human behaviour in malaria transmission in a highland region of the Republic of Yemen
Source: Malar J. 2016 Mar 1;15:130. doi: 10.1186/s12936-016-1179-8 (PMC4774125; doi:10.1186/s12936-016-1179-8)
Supplement: Supplementary file 1 — 10.1186/s12936-016-1179-8 House survey and knowledge, attitudes and perception (KAP) survey. [file 12936_2016_1179_MOESM1_ESM.pdf]

Entomological aspects and the role of human behaviour in malaria transmission in a highland region of the Republic of Yemen S. M.A. Al-Eryani *et al.*

SUPPLEMENT 1 **House survey and knowledge, attitudes and perception (KAP) survey**

Greetings. I am doing health research in this community. I am trying to learn more about health problems here, and am very grateful to you for agreeing to talk with me. May I now ask you a few questions.

السلام عليكم,
أنا أقوم بعمل بحث عن الصحة. و أحاول أن أعرف ما هي مشاكلكم الصحية و أنا شاكر لكم موافقتكم على العمل معي. فهل بالإمكان أن أسألكم بعض الأسئلة؟

Questionnaire

استبيان

Malaria survey Taiz-Yemen

مسح مالا ريا: تعز- اليمن

District:

المدرية:

ID No:

رقم التعريف:

Village name

اسم القرية:

Date (dd/mm/yyyy)

التاريخ:

Time started:

وقت البدء:

Time ended:

وقت الانتهاء:

Background information
المعلومات
الخاصة

Name of respondent

الاسم:

....

Age:

العمر:

. ...

Sex: Male/Female

الجنس: ذكر\أنثى

Marital status: married/widowed/single/divorced

الحالة الاجتماعية:متزوج\أرمل\عازب\مطلق

1. How many people sleep in this house? [ ]

1- كم شخص يعيش في المنزل؟ [ ]

2-Have you ever attended school?
Yes/No

2-هل سبق لك الذهاب إلى المدرسة؟
نعم\لا

If YES, what was the last grade you completed? [ ]

إذا نعم, ماذا كانت أخر مرحله لك؟ ( )

(O=Not attended/Illiterate; 1=Elementary; 2=Secondary; 3=University 4=Higher studies)

(صفر=غير متعلم 1=أساسي 2=ثانوي 3=جامعي 4=دراسات عليا)

3-Is malaria a problem for your household?

3-هل الملا ريا مشكله في عائلتك؟

(1=Yes; 2=No; 3=Don't know)

(1=نعم 2=لا 3=لا أعلم)

4- How can you get Malaria?

4--كيف يمكنك الاصابه بالمالاريا؟

5-.What time of year is malaria a problem? (Wet, dry, all year, don't know)

5-. أي وقت في السنة تكون الملا ريا مشكله؟ (الرطوبة, طول السنة, لا أعرف)

6-Mentioned mosquitoes [ ]

6-هل ذكر بإجابتك البعوض(الناموس) ( )

('1=Yes; 2=No; 3=No answer)

(1=نعم, 2=لا, 3=لم يجب)

7-.How do you know someone has malaria?

7.-كيف تعرف أن الشخص مصاب بالملا ريا؟

(Please tick)

(أشر هنا)

1-Hot head

1-سخونة الرأس

2-Hot body

2-سخونة الجسم

3-Diarrhea

3-إسهال

4-Coma

4-غيبوبة

5-Dizziness

5-دوار

6-Cough/chest

6-سعال

7-Death

7-الوفاة

8-Vomiting

8-اقياء

9-Yellow eyes

9-صفار العينين

10-Constipation

10-إمساك

11-Fever

11-حمى

12-Weakness

12-ضعف

13-Lethargic

13-إجهاد

14-Muscle pain

14-الم في العضلات

15-Joint pain

15-الم في المفاصل

16-Headache

16-صداع

17-Sleepy

17-نعاس

18-Can't sleep

18-أرق

19-Body pain

19-الم في الجسم

20-Cold sweat

20-عرق بارد

21-Urine color
(yellow, red)

21-لون البول
(أصفر, أحمر)

Don’t know

لا أعرف

If Other, please specify

أخرى, أذكر

8-.Can you prevent yourself (or your household) from getting malaria?
(Write Yes/No)

8-هل يمكنك حماية نفسك (أو أسرتك) من الاصابه بالملا ريا؟
(أكتب نعم\لا)

8a. If Yes, how?

8 أ-. إذا نعم, كيف؟

....

<u>Mosquitoes</u>

<u>الناموس</u>

9-.Where do mosquitoes come from?

9.- من أين يأتي البعوض؟

....

10-.What time of the day do they mostly bite?
Please tick:

10 - في أي وقت باليوم يعض البعوض؟
يرجى الإشارة:

-Day

-النهار

-Night (early or late)

-الليل (مغرب أو أخر الليل)

-All day

-طوال اليوم

-Don't know

-لا أعرف

11-What time of year do they mostly bite?
Please tick:

11- في أي وقت بالسنة يعض البعوض؟
يرجى الإشارة:

-Wet

-الرطوبة

-Dry

-الجفاف

-All year

-طوال السنة

-Don't know

-لا أعرف

12-.Do you know of any diseases transmitted by mosquitoes?
.... [ ]

12- هل تعرف أي مرض أخر ينتقل بواسطة البعوض؟
[ ]..

13-.Do you sit outside during the evenings?

13-هل تجلس خارج المنزل خلال الليل؟

14-.Are the mosquitoes a problem for you and your households?
Please tick:

14-هل البعوض مشكله بالنسبة لك و لعائلتك؟
يرجى الإشارة:

Yes

نعم

No

لا

Don't know

لا أعرف

15-.Do you protect yourself from mosquitoes?
Please tick:

15.هل تحمي نفسك من البعوض؟
يرجى الإشارة:

Yes

نعم

No

لا

Don't know

لا أعرف

15a. If YES fill in the table:

15 أ. إذا نعم, أملأ الجدول:

Ask to see net, status, if impregnated.

اطلب رؤية الناموسية وحالتها وإذا كانت معالجه

Trap doors/ nettings for windows? أبواب مشتبكة أو شبك على النوافذ؟		All or Bedroom? جميع الغرف أو غرفة النوم؟	
Bed net ناموسية		Who sleeps under? من ينام تحت الناموسية؟	
Coil أبخرة		How often used and how many? متى تستعمل وكم الكمية؟	
Burn herbs حرق أعشاب		How often? متى تستعمل؟	
Spray بخاخ		How often? متى تستعمل؟	
Others أخرى		Specify حدد	

.

16-If they do not use a bed net, please ask the following questions:

16. إذا كانت الناموسية لا تستعمل, اسأل الأسئلة التالية:

a. Why do you not use a bed net?

أ.لماذا لا تستعمل الناموسية؟

....

If mentioned costs in (a.) Please ask:

إذا ذكر بإجابة (أ) أن السبب هو التكلفة, اسأل:

b-Are there, apart from costs, other reasons for not using a bed net?

هل هناك سبب أخر غير التكلفة؟

....

c. Do you know where you can obtain a bed net?

ج.هل تعرف أين يمكنك الحصول على الناموسية؟

Yes/No

نعم\لا

If Yes. Where?

إذا نعم, أين؟

....

d. If you were given a bed net, who would sleep under it?

د. اذا أعطيت ناموسية, من سوف ينام تحتها؟

Code: Head of household 1; Wife 2; Children 3

رب المنزل 1, الزوجة 2, الأطفال 3.

....

.

17-Have you, or anyone in your house, had malaria recently?
(please write Yes/ No)

17- هل أصبت أنت أو أحد من أفراد الأسرة بالملا ريا مؤخرا؟
(أجب نعم\لا)

a. If Yes, How do you know it was malaria?
(Please tick)

symptoms tell

من الأعراض

doctors judgment

تشخيص الطبيب

other, specify

أخرى, حدد

....

b. What did you do?
(Please tick)

ب. ماذا فعلت؟
(الرجاء الاشاره)

-To clinic

-ذهبت إلى العيادة

-Bought drugs

-اشتريت الدواء

-Nothing

-لا شيء

other, please specify

أخرى, حدد

……………………………………………

C. If visited a clinic, was a blood film made? (Yes/ No)

ج. إذا ذهبت إلى العيادة فهل أجريت فحص الدم؟ (نعم\لا)

d. If drugs were prescribed, what did you take?
(Please tick)

د. إذا كنت اشتريت دواء, ما هو؟

–Chloroquine

-كلوروكوين

–Fansidar

-فانيسيدار

-paracetamol

-باراسيتامول

Artesunate

-ارتسونيت

-Other, specify

-أخرى, حدد

e. Where did you get the drugs?
(Please tick)

ه. من أين حصلت على الدواء (ضع اشاره)

doctors prescription at clinic/pharmacy

الصيدلية أو العيادة حسب وصفة الطبيب

At pharmacy without a prescription.

الصيدلية بدون وصفة الطبيب

Other, specify

أخرى, حدد

That's all I wish to ask you. Thank you very much for your help. Is there anything you would like to ask me now?

هذا كل ما أريد أن سؤالكم. شكرا لمساعدتكم.
هل لديكم أسئلة تريدون طرحها؟

Name of Interviewer:

اسم الذي أجرى المقابلة:

………………………………………………………………………………………………
.
